# L‐quebrachitol Modulates the Anti‐convulsant Effects of Carbamazepine Possibly Through Voltage‐gated Sodium Channel Blocking Mechanism in Chicks: In Vivo and In Silico Studies

**DOI:** 10.1002/brb3.70675

**Published:** 2025-07-07

**Authors:** Asifa Asrafi, Mohammad Aslam, Ali G. Alkhathami, Md. Sakib Hossain, Imam Hossen Rakib, Md. Sakib Al Hasan, Feroz Khan Nun, Md. Faisal Amin, Muhammad Torequl Islam

**Affiliations:** ^1^ Department of Biochemistry and Molecular Biology Gopalganj Science and Technology University Gopalganj Bangladesh; ^2^ Department of Pharmacy Gopalganj Science and Technology University Gopalganj Bangladesh; ^3^ Bioinformatics and Drug Innovation Laboratory BioLuster Research Center Ltd. Gopalganj Bangladesh; ^4^ Department of Clinical Laboratory Sciences, College of Applied Medical Sciences King Khalid University Abha Saudi Arabia; ^5^ School of Integrative Biological and Chemical Sciences The University of Texas Rio Grande Valley Texas USA; ^6^ Pharmacy Discipline Khulna University Khulna Bangladesh

**Keywords:** convulsion, L‐Quebrachitol, molecular docking study, sodium channel

## Abstract

**Introduction:**

L‐Quebrachitol (LQB), a naturally occurring bioactive compound, exhibits anti‐inflammatory, anti‐oxidant, anti‐cancer, and anti‐diabetic properties. However, its therapeutic potential in convulsant management remains largely unexplored. The objective of this study was to investigate the anticonvulsant effects of LQB in an In Vivo model and to examine its molecular interactions via In Silico docking simulations.

**Methods:**

In the In Vivo study, pentylenetetrazol (PTZ) was administered intraperitoneally (i.p.) at 80 mg/kg to induce convulsions, and the test animals were treated orally with three doses of LQB (1, 5, and 10 mg/kg), with carbamazepine (CBZ) at 80 mg/kg as a standard drug.

**Results:**

The results indicated that LQB at all tested doses significantly (*p* < 0.05) prolonged seizure latency and decreased convulsion frequency, with the 10 mg/kg dose showing the most significant effects. Furthermore, the combination of LQB (10 mg/kg) and CBZ (80 mg/kg) resulted in a synergistic increase in anticonvulsant activity. In the In Silico study, molecular docking analysis revealed that both LQB and CBZ interacted with the voltage‐gated sodium channel (VGSC), a key receptor involved in convulsions, with LQB demonstrating a binding affinity (BA) of −5.4 kcal/mol, which was moderate compared to CBZ's BA.

**Conclusion:**

LQB showed potential anti‐convulsant activity in PTZ‐induced convulsion animals, possibly through blocking sodium channel receptors. Further studies are needed to clarify its mechanisms and clinical potential in convulsion treatment.

## Graphical abstract

L‐quebrachitol exhibited significant anticonvulsant effects in PTZ‐induced seizures in chicks, likely through voltage‐gated sodium channel inhibition. In vivo and in silico analyses suggest LQB may synergize with carbamazepine, offering a potential new therapeutic approach for epilepsy management.

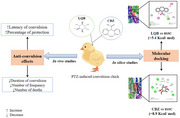

**Abbreviations**
%P: Percentage protection.
BA: binding affinity
CBZ: carbamazepine
DC: Duration of convulsion
HB: hydrogen bond
i.p.: intraperitoneally
LC: Latency of convulsion
LQB: L‐Quebrachitol
ND: Number of deaths
NF: Number of frequency
PTZ: pentylenetetrazol
VGSC: voltage‐gated sodium channel

## 1 Introduction

Epilepsy is one of the most common neurological and highly heterogeneous clinical conditions, affecting approximately 1% of the global population (Balestrini et al. 2021; Shan et al. 2024). It is defined as repeated, unprovoked seizures caused by many medical, psychological, and social factors. High electrical discharge in brain cells causes recurrent seizures that lead to epilepsy (Alhashimi et al. 2022). Epilepsy is also among the causes of death around the globe, thereby being associated with about 125,000 deaths annually, which is so alarming (Trinka et al. 2023). More than 80% of these deaths are in the low‐ and middle‐income countries. The number of people reaching total seizure control has stayed constant despite the development of more than 20 new anti‐seizure medications in recent decades (Yu et al. 2021).

Voltage‐gated and ligand‐gated ion channels are studied in relation to their function in the development of novel antiepileptic drugs (AEDs) and in the epileptogenesis of both inherited and acquired epilepsies (Armijo et al. 2005). VGSCs are integral to the propagation of action potentials in neurons; their dysfunction and genetic mutation play a pivotal role in the development and management of epilepsy (Ademuwagun et al. 2021). However, it is important to note that VGSCs are not only involved in neuronal signaling but also contribute to various physiological functions such as cardiac rhythm and gastrointestinal motility (Coates et al. 2019; George 2005; Yuill and Smirnov 2010; Lu et al. 2022). Non‐selective VGSC inhibition may lead to systemic adverse effects, including impaired peristalsis and autonomic dysfunctions (Bielefeldt and Bass 1991; Regan et al. 2024). Therefore, compounds targeting VGSCs for epilepsy treatment must be evaluated for selectivity to minimize off‐target risks.

CBZ, lamotrigine, and phenytoin primarily work by blocking Na^+^ channels, which appears to be how traditional and innovative AEDs work (Rana et al. 2023). Epilepsy, characterized by recurrent seizures, is often linked to abnormalities in VGSC function. Genetic mutations, altered expression, or dysregulated gating dynamics could be the cause of these abnormalities. Mutations in genes encoding VGSCs, such as SCN1A, SCN2A, and SCN8A, play a vital role in epilepsy (Rusina et al. 2024). SCN1A mutations are commonly linked to Dravet Syndrome, a severe epileptic encephalopathy characterized by cognitive deficits and early‐onset seizures (Sadleir et al. 2017). SCN2A mutations change the voltage dependency of channel activation and inactivation, producing age‐dependent epilepsy (Hedrich et al. 2019). These mutations are frequently linked to benign familial neonatal‐infantile seizures (BFNIS).

Natural products are biologically active compounds from living organisms that serve as key sources or templates for drug development (Aktar et al. 2024; Bithi et al. 2025). Natural products and their derivatives may be useful for the treatment of epilepsy because of their presumed anticonvulsant activity and fewer adverse effects than standard AEDs (Challal et al. 2023). Interestingly, substances that occur naturally and their derivatives are valuable in cases of epilepsy by reducing free radicals and stimulating neuroprotective pathways, more notably NRF2 (nuclear factor erythroid 2‐related factor 2) (Firdous et al. 2025). Flavonoids and terpenoids present in medicinal plants used for epilepsy help to reduce oxidant levels, which is the main cause of the disease (He et al. 2021). Phenylpropanoids, in contrast, have been identified as potential multitarget compounds with relevant actions on significant neurotransmitter systems, including GABA_A_ and AMPA, together with reasonable pharmacokinetics (Rodrigues et al. 2024). In animals, the natural organic compounds include cinnamon, Nigella sativa, and curcumin, which have been shown to enhance cognition in animals, reduce neuroinflammation, and decrease seizure frequency and severity in animals when used by animals (Grabarczyk et al. 2024). These products enhance neuronal function and act on neurotransmitter systems regulating seizures through a reduction of inflammation and promotion of neuroplasticity in the GABAergic and glutamatergic systems. Another research article indicates that curcumin suppresses the inflammation NF‐κB signaling pathway and enhances the BDNF protein, which promotes neuronal development and synapse repair (Choi et al. 2017).

LQB, also known as 2‐O‐Methyl‐L‐chiro‐inositol, is a naturally occurring cyclitol substance that is extracted from *Aspidosperma quebracho* bark and has a variety of biological applications in materials science and medicine (Wu et al. 2016). A major source of LQB is sea buckthorn, scientifically known as *Hippophae rhamnoides* (Żuchowski 2023). LQB is a methylated inositol white, crystalline substance that has many biological properties, such as anti‐inflammatory, anti‐oxidant, anti‐cancer, anti‐diabetic, and anti‐platelet aggregation properties (Li et al. 2023; Yodthong et al. 2018). Studies indicate that inositol and its derivatives have neuroprotective activity (Ali et al. 2022). Chiro‐inositols, including 2‐O‐Methyl‐L‐chiro‐inositol, act as insulin mimetics. They enhance insulin signaling in the central nervous system, which is crucial for protecting synapses from damage caused by toxic Aβ oligomers associated with Alzheimer's disease (Pitt et al. 2013). Previous studies show that inositol and methyl inositol have free radical scavenging properties (López‐Gambero et al. 2020; Yang et al. 2011). LQB promotes protection in neurological treatments, particularly for neurodegenerative illnesses. A previous study shows that LQB has great potential in the treatment of neurological diseases and disorders due to its anti‐oxidant properties (de Araújo and de Barros Viana 2020). Possibly stabilizing cell membranes and scavenging free radicals, LQB reduces oxidative damage, a major contributing cause to neurodegenerative diseases. In studies using rat fetal mesencephalic cell cultures, quebrachitol demonstrated protection against cytotoxicity induced by 6‐hydroxydopamine (6‐OHDA), a substance that resembles the oxidative stress seen in neurodegenerative diseases (Junior et al. 2006; Wu et al. 2016). Another study indicates that methyl‐inositols are bioactive components regulating sugar metabolism and protecting cells from oxidative, cytotoxic, and mutagenic damages (de Olinda et al. 2008; Kallio et al. 2009). Because of its ability to protect neuronal cells from oxidative stress and cell death, our findings and test experiment suggest LQB could be a viable option for the creation of therapeutic medications for neurodegenerative illnesses.

This study aimed to evaluate the anticonvulsant potential of LQB using a PTZ‐induced seizure model In Vivo and to explore its VGSC blocking mechanism through In Silico studies.

## 2 Materials and Methods

### 2.1 In Vivo Studies

#### 2.1.1 Chemicals and Reagents

LQB (CAS: 642‐38‐6, Purity: ≥ 98.5% (HPLC)) and PTZ (CAS: 54‐95‐5, Purity: ≥ 99% (GC)) were purchased from Sigma Aldrich (Germany), while CBZ was kindly supplied by Square Pharmaceuticals Ltd. (Bangladesh). Tween 80 and sodium chloride (NaCl) required for this study were purchased from Merck (India).

#### 2.1.2 Experimental Animals

Young broiler chicks (*Gallus domesticus*) of either sex, with a body weight range of 38–42 g, 2 days old, were purchased from a local market in Khulna, Bangladesh. The chicks were acclimatized for 2 days at the pharmacology lab of Gopalganj Science and Technology University (GSTU) before starting this study. During this time the chicks had free access to standard foods and water ad libitum. Room temperature was maintained at 27 ± 2°C with a 12‐h dark/light cycle under controlled illumination. After 12 h of fasting, this study was carried out. However, they were allowed free access to water only. Studies were performed between 9:00 a.m. and 3:00 p.m. This study was approved by the Animal Ethics Committee of Khulna University (KUAEC‐2024‐03‐01).

#### 2.1.3 Dose Selection and Study Design

In this study, we used 1, 5, and 10 mg/kg doses in chicks. The test doses for LQL (1, 5, and 10 mg/kg) were selected according to a previous study by Yodthong et al. (2018). For this, we have randomly divided thirty animals into six treatment groups. A control (vehicle: distilled water containing 0.9% NaCl and 0.5% Tween 80), a standard group consisting of CBZ at 80 mg/kg, and three test doses (LQB) were treated, respectively, at 10 mL/kg. Finally, the sixth group was combined with CBZ‐80 and LQB‐10. PTZ was treated after five minutes of treatment groups. PTZ treatment details are provided below. Before treatment, all animals were fasted overnight. The study design section (Table 1) shows the animal grouping and treatment protocol.

**TABLE 1:** Treatment groups with their details at 10 mL/kg volume of oral administration.

Treatment groups	Description
Control	Vehicle: Distilled water containing 0.9% NaCl and 0.5% tween 80
CBZ‐80	Carbamazepine (sodium channel blocking reference drug) at 80 mg/kg
LQB‐1	L‐Quebrachitol (test sample) at 1 mg/kg
LQB‐5	L‐Quebrachitol (test sample) at 5 mg/kg
LQB‐10	L‐Quebrachitol (test sample) at 10 mg/kg
CBZ‐80+LQB‐10	Carbamazepine 80 mg/kg + L‐Quebrachitol 10 mg/kg

***Note***: Each group contains five animals.

**Abbreviations**: CBZ, carbamazepine; Control, vehicle (distilled water containing 0.9% NaCl and 0.5% tween 80); LQB, l‐quebrachitol.

#### 2.1.4 Pentylenetetrazol (PTZ)‐induced Convulsion Test

This study was done according to the method described by Herrera‐Calderon et al. (2017). Thirty minutes after all treatments, PTZ at a dose of 80 mg/kg was administered intraperitoneally (i.p.) (Herrera‐Calderon et al. 2017; Islam et al. 2025). Then the first convulsion time, denoted as the latency of convulsion (LC), number of frequency (NF), and duration of convulsion (DC), along with the percentage of deaths (%D), were recorded. The percentage protection (%) was calculated by using the following formula:

$$\begin{array}{ccc} \%\;\mathrm{Protection} & = & \left[\left(\mathrm{Control}\;\mathrm{Death}\;-\mathrm{Test}\;\mathrm{Death}\right)÷\mathrm{Control}\;\mathrm{Death}\right]\\ & & \times 100 \end{array}$$

#### 2.1.5 Statistical Analysis

Values are expressed as the mean ± SEM (standard error of the mean). One‐way ANOVA (analysis of variance) followed by *t*‐students *Tukey post‐hoc* test with multiple comparisons at 95% confidence intervals using GraphPad Prism software (version: 9.5, San Diego, USA). Data were considered significant when *p <* 0.05.

### 2.2 In Silico Studies

#### 2.2.1 Ligand Preparation

**FIGURE 1:**
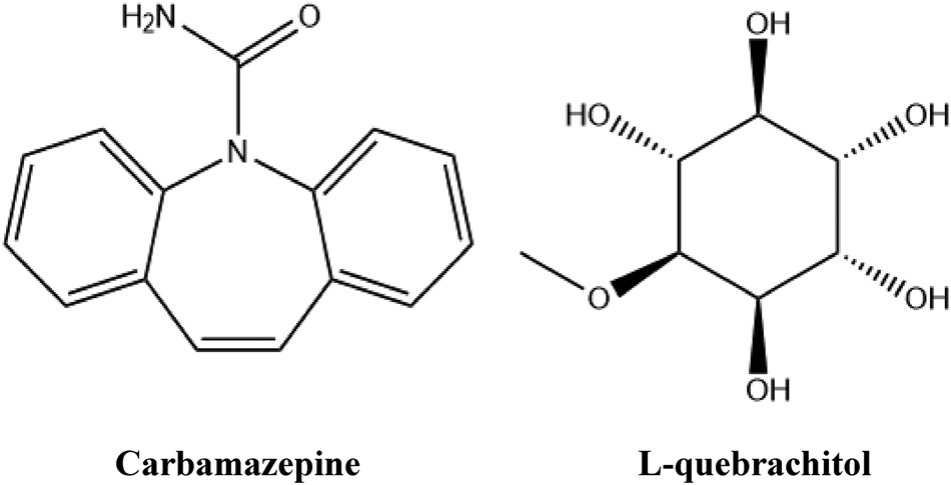
The two‐dimensional chemical structures of LQB and the referral drug CBZ.

The 3D structures of CBZ (PubChem CID: 2554) and LQB (PubChem CID: 151108) were downloaded in SDF format from the PubChem online chemical database (https://pubchem.ncbi.nlm.nih.gov/, received on January 30, 2025). To minimize these chemical agents, we used Allinger's force field (MM2) method by using the Chem3D 16.0 program package (Ferdous et al. 2024). Figure 1 shows 2D structural representations of the chemical compounds.

#### 2.2.2 Protein Selection and Preparation

According to a recently published paper, we selected VGSC receptors that are responsible for convulsions (Chowdhury et al. 2024). The 3D structure of VGSCs (PDB ID: 8S9C and chain: (A) was obtained from the RCSB Protein Data Bank (https://www.rcsb.org/, received on January 30, 2025). To optimize the receptor by eliminating unwanted protein chains and external components such as water molecules, lipids, and heteroatoms by using PyMol software (v2.4.1) (Islam et al. 2024). Eventually, we used Swiss‐PDB viewer software (version 4.1.0) to minimize the energy of the protein structure by modifying the GROMOS96 43 B1 force field (Jahan et al. 2025).

#### 2.2.3 Molecular Docking Protocol

After preparing ligands and receptors, we used PyRx software to perform the molecular docking process to predict how well potential binding of the ligands against the active site of the receptors would occur (Yadav et al. 2024). The docking process involved grid box measurements (along the x‐, y‐, and z‐axes were fixed to 85 × 80 × 75 Å, and the calculation was run in 2000 steps). The result of the docking was saved in CSV format, and the best pose was saved in PDBQT format. For analyzing non‐bond interactions, including amino acid residues, bond length, type of hydrogen bonds (HBs), and other bonds, we used Discovery Studio Visualizer (v21.1.020298) (Al Hasan et al. 2024; Al Hasan et al. 2025).

## 3 Results

### 3.1 In Vivo Study

#### 3.1.1 PTZ‐induced Convulsion Study

According to our analysis of the In Vivo study, we observed that all animal groups exhibited 100% induction of convulsion following the administration of PTZ. The negative control (NC) group received distilled water containing 0.9% NaCl and 0.5% Tween 80 and acted as a baseline for comparison, exhibiting the most severe convulsion episodes, characterized by rapid onset (19.20 ± 1.17 s), with 100% mortality shown in the NC group. However, the established anticonvulsant drug (CBZ‐80) significantly (*p* < 0.05) delayed the onset of convulsion (113.40 ± 5.78 s) when compared to an NC group. Different groups of test samples (LQB) produce dose‐dependent impacts on the PTZ‐induced animals and significantly (*p* < 0.05) increase LC compared to the NC group. LQB‐1, LQB‐5, and LQB‐10 animal groups demonstrated latency of convulsion at 22.40 ± 1.32, 36.60 ± 4.62, and 62.20 ± 4.31 s. When a combination of a high dose of a test sample and a standard drug (CBZ‐80+LQB‐10) was used, we observed a synergistic effect. The combination group significantly (*p* < 0.05) delayed the onset of convulsions (137.40 ± 3.50), which was higher than the CBZ‐80.

In the case of NF and DC, we observed brief vibration with a duration of one to three seconds, but no indications of seizures that lasted longer. The NC group exhibited high frequency (74.20 ± 3.18) and prolonged duration (372.40 ± 11.27 s). On the other hand, CBZ‐80 showed a shortened frequency (12.40 ± 1.70) and duration (86.40 ± 5.37 s), resulting in protecting 80% of animals, which was lower than the NC group. LQB exhibited dose‐dependent and significant (*p* < 0.05) reduction in both NF and DC. LQB‐10 significantly (*p* < 0.05) reduced NF (15.20 ± 1.43) and DC (127.20 ± 3.52 s) compared to LQB‐1 and LQB‐5. As a result, 80% of a animals were saved from PTZ toxicity and prevented from death, which was similar to the standard drug (CBZ‐80) and the combination drug (CBZ‐80+LQB‐10). Finally, the combination group (CBZ‐80+LQB‐10) revealed the lowest NF (10.00 ± 1.00) and DC (74.80 ± 1.56 s) compared to other groups*. In vivo* results are shown in Table 2.

**TABLE 2:** Convulsion parameters observed in various treatment groups.

Treatment groups	LC	NF	DC	%D	%P
Control	19.20 ± 1.17	74.20 ± 3.18	372.40 ± 11.27	100.00 ± 0.00	0
CBZ‐80	113.40 ± 5.78^*bcd^	12.40 ± 1.70^*bcd^	86.40 ± 5.37^*bcd^	20.00 ± 0.18^*bc^	80
LQB‐1	22.40 ± 1.32^*^	32.80 ± 3.38^*^	207.60 ± 3.94^*^	60.00 ± 0.24^*^	40
LQB**‐5**	36.60 ± 4.62^*b^	21.20 ± 0.73^*b^	181.60 ± 3.24^*b^	40.00 ± 0.24^*b^	60
LQB‐10	62.20 ± 4.31^*bc^	15.20 ± 1.43^*bc^	127.20 ± 3.52^*bc^	20.00 ± 0.20^*bc^	80
CBZ‐80+LQB‐10	137.40 ± 3.50^*abcd^	10.00 ± 1.00^*abcd^	74.80 ± 1.56^*abcd^	20.00 ± 0.18^*bc^	80

***Note***: Values are mean ± SEM (*n* = 5); One‐way ANOVA followed by *t*‐students *Tukey post‐hoc* test with multiple comparisons; *p <* 0.05 compared to the *Control, ^a^CBZ‐80, ^b^LQB‐1, ^c^LQB‐5, and ^d^LQB‐10.

**Abbreviations**: CBZ, carbamazepine; Control, vehicle (distilled water containing 0.9% NaCl and 0.5% tween 80); DC, duration of convulsion; LC, latency of convulsion; LQB, L‐Quebrachitol.; ND, number of deaths; NF, number of frequencies; %P, percentage protection.

#### 3.1.2 Tolerability and Gastrointestinal Observation

Throughout the study period, no signs of gastrointestinal abnormalities, including diarrhea or altered stool frequency, were observed in any of the LQB‐treated chick groups. Animals maintained normal behavior, feeding, and water consumption. The absence of gastrointestinal side effects suggests a favorable short‐term safety profile of LQB at the tested doses (1, 5, and 10 mg/kg). However, extended duration studies are recommended to further confirm these findings.

### 3.2 In Silico Findings

#### 3.2.1 LQB and CBZ With Voltage‐gated Sodium Channel Receptor Interactions

**FIGURE 2:**
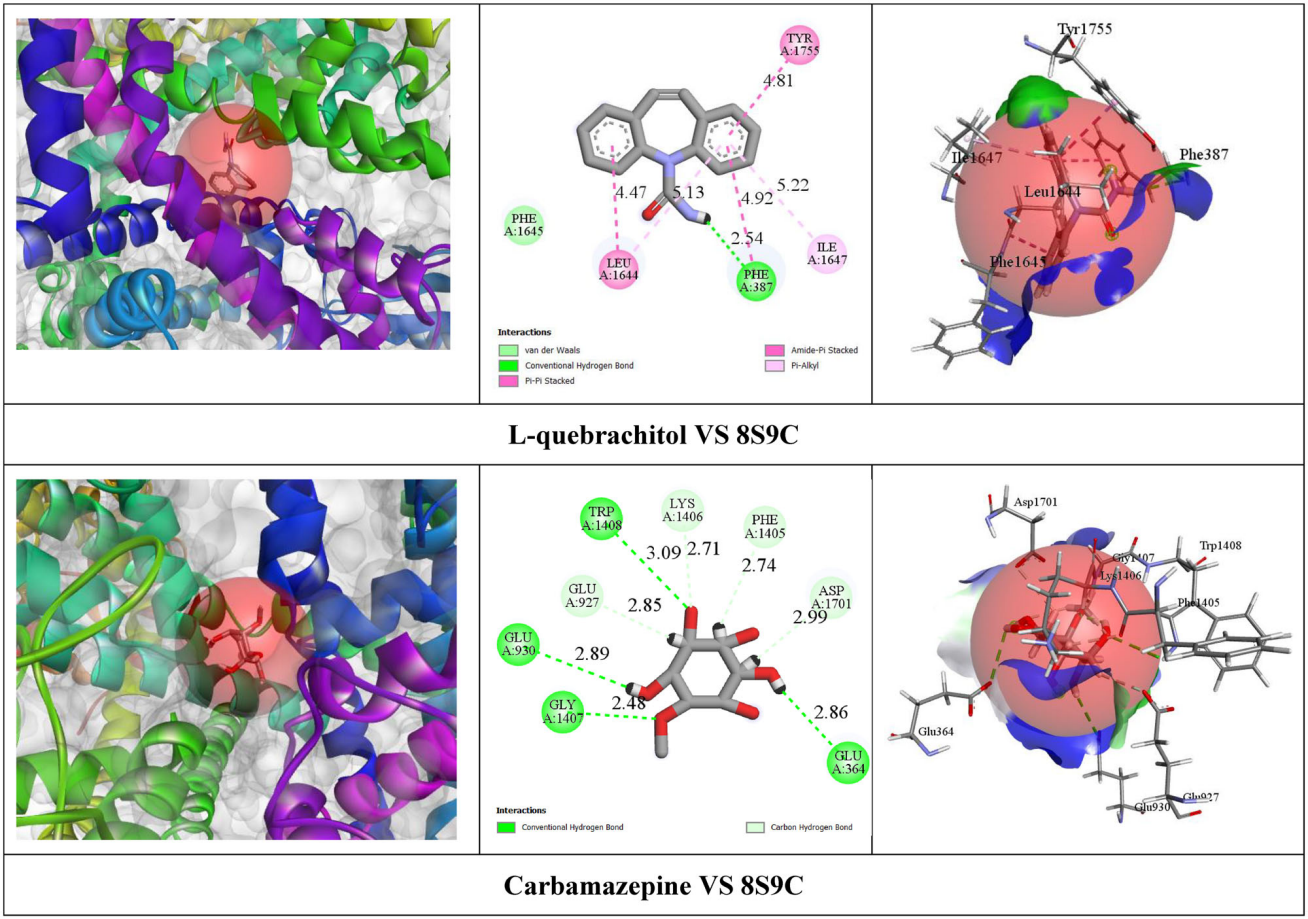
The two‐dimensional and three‐dimensional non‐bond interaction of CBZand LQB with the voltage‐gated sodium channel receptor (8S9C).

According to the In Silico study, CBZ exhibited relatively higher BA (−8.9 kcal/mol) with the VGSC receptor by the formation of one HB with PHE A: 387 (2.54 Å) amino acid (AA) residue. Additionally, CBZ formed four hydrophobic (HP) interactions with specific AA residues, including PHE A: 387, TYR A: 1755, LEU A: 1644, and ILE A: 1647. On the other hand, LQB demonstrated moderate BA (−5.4 kcal/mol) against the VGSC receptor by the formation of eight HBs with GLY A:1407 (2.48 Å), TRP A:1408 (3.09 Å), GLU A:930 (2.89 Å), GLU A:364 (2.85 Å), LYS A:1406 (2.70 Å), ASP A:1701 (2.99 Å), GLU A:927 (2.84 Å), and PHE A:1405 (2.73 Å) specific AA residues. Table 3 shows BA, HBs, and other relevant AA residues involved in the interactions of LQB and CBZ with the VGSC receptor. Figure 2 showed the 2D and 3D nonbond interactions of LQB and CBZ with the VGSC receptor.

**TABLE 3:** The binding affinity and non‐bond interactions of CBZ and LQB.

Ligands	Proteins (PDB ID)	Protein chain	BA (kcal/mol)	AA residues		
				HB amino residues	HB length (Å)	Others bonding amino residues
LQB	8S9C	A	−5.4	GLY A: 1407 TRP A: 1408 GLU A: 930 GLU A: 364 LYS A: 1406 ASP A: 1701 GLU A: 927 PHE A: 1405	2.48 3.09 2.89 2.85 2.70 2.99 2.84 2.73	—
CBZ			−8.9	PHE A: 387	2.54	PHE A: 387 TYR A: 1755 LEU A: 1644 ILE A: 1647

**Abbreviations**: AA, amino acid.; BA, binding affinity; CBZ, carbamazepine; HB, hydrogen bond; LQB, l‐quebrachitol; PDB ID, protein data bank identifier; VGSC, voltage‐gated sodium channel.

## 4 Discussion

VGSC receptor responsible for the management of several central nervous system diseases, including epilepsy, chronic pain, psychiatric disorders, and spasticity. Because it regulates the excitability and physiological process (Pal et al. 2021). Established AEDs such as CBZ, phenytoin, lamotrigine, oxcarbazepine, rufinamide, and lacosamide suppress abnormal epileptiform activity by blocking the VGSC (Brodie 2017). However, long‐term use of CBZ can cause leukopenia, drug eruptions, hyponatremia, disturbances of vitamin D metabolism, and hepatitis (Huang et al. 2024). Use of phenytoin can cause several side effects, including hallucinations, confusion, hypotension, hyperactivity, megaloblastic anemia, cardiovascular collapse, and peripheral neuropathy, leading to withdrawal symptoms (Faturachman et al. 2022). In 2017, AED retigabine was withdrawn from the market due to ophthalmological and dermatological factors (Brickel et al. 2020). Consequently, currently available AEDs have limitations in terms of safety, and improved AED safety profiles and urgently needed discovery of new medication as soon as possible. Many AEDs are derived from natural products such as plants and herbs (Bhardwaj et al. 2022). Cannabidiol, a natural compound found in cannabis, has recently gained attention as a potential treatment for seizures and demonstrated outstanding results (Singh et al. 2023; Talwar et al. 2023). Also, curcumin (Forouzanfar et al. 2021), resveratrol (Ethemoglu et al. 2017), and valerian root (González‐Trujano et al. 2021) show promise in clinical studies, but further research is needed to fully understand their potential, and natural sources can be significant resources for discovering new medications.

PTZ is a commonly used convulsant that induces seizures by increasing neuronal excitability, possibly through interactions with sodium voltage‐gated ion channels (Samokhina and Samokhin 2018). In our experiment, PTZ injection led to the rapid onset of seizures in the control group, accompanied by a high frequency of convulsions. LQB pretreatment delayed seizure onset, reduced seizure frequency and duration, and improved survival rates in a dose‐dependent manner. These findings suggest that LQB may provide neuroprotective effects against PTZ‐induced seizures.

Our study revealed that LQB‐10 significantly (*p* < 0.05) enhanced seizure latency, reduced convulsion frequency, and shortened seizure duration, indicating strong anticonvulsant activity. Notably, the CBZ‐80+LQB‐10 combination showed the most potent effects, suggesting a synergistic interaction that improves seizure control more effectively than either agent alone (Yi et al. 2022). Among all tested doses, LQB at 10 mg/kg exhibited the most significant anticonvulsant activity, evident through delayed seizure onset, reduced frequency, and shortened duration. Notably, its co‐administration with CBZ enhanced these effects, suggesting a synergistic interaction likely mediated via VGSC inhibition. These findings highlight the therapeutic potential of LQB while also emphasizing the importance of targeting drug delivery selectively to the brain to minimize peripheral VGSC‐related adverse effects.

**FIGURE 3:**
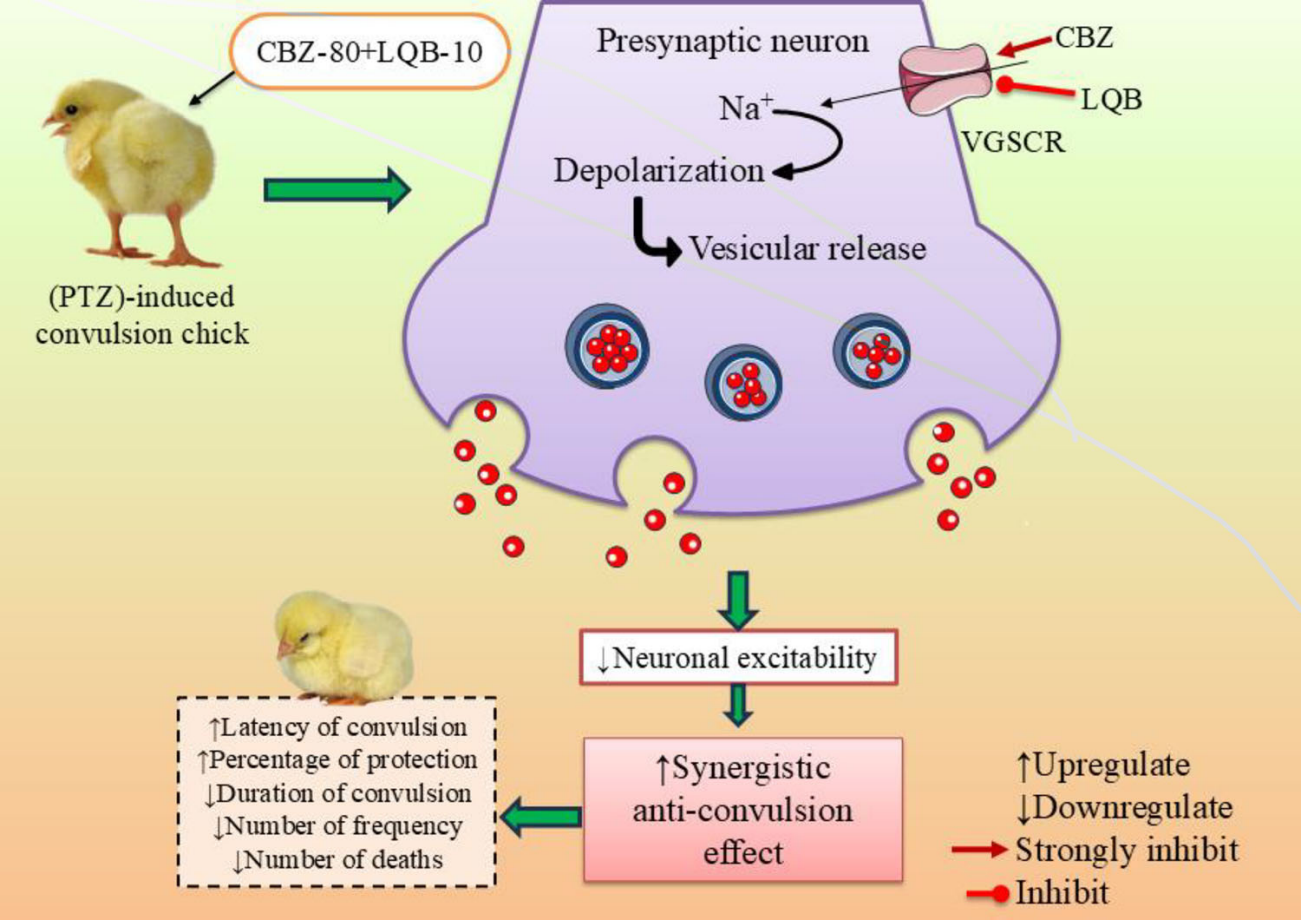
The possible anti‐convulsion mechanism of LQB [The diagram illustrates the synergistic anti‐convulsant mechanism of CBZ) and LQB‐10 in PTZ (pentylenetetrazole)‐induced convulsion in chicks. CBZ and LQB‐10 strongly inhibit VGSCs at the presynaptic neuron, reducing Na⁺ influx and neuronal depolarization, which leads to decreased vesicular neurotransmitter release and lowered neuronal excitability. This combined action results in a synergistic anti‐convulsion effect, marked by increased latency to convulsion and protection percentage, along with reduced duration and frequency of convulsions and lower mortality.

However, the possible convulsion mechanism of LQB is shown in Figure 3.

Computer‐aided drug design (CADD) techniques are more significant in reducing the reliance on animal models in pharmacological research and assessing more chemicals in a short time with lower cost. The methodology aids in the exploration and analysis of natural products with promising qualities as well as the development of novel compounds by medicinal chemists and pharmacologists (Rahman et al. 2022; Chowdhury et al. 2024). Use of In Silico studies also helps to understand the metabolic pathways of these active molecules (Saeidnia et al. 2013). However, In Silico studies have some limitations, including validity and reliability, complexity of molecular dynamics, limited diversity of molecules tested, and scoring functions and algorithms (https://proventainternational.com/a‐critical‐evaluation‐of‐the‐advantages‐and‐limitations‐of‐in‐silico‐methods‐in‐clinical‐research/). Despite these drawbacks, In Silico techniques have enormous potential to improve our comprehension of neurological conditions and create efficient therapies. In our In Silico study, LQB provides moderate effects compared to the standard drug (−5.4 kcal/mol), so future research should focus on optimizing LQB's structure, conducting molecular dynamics simulations, and validating its efficacy through in vitro and In Vivo studies.

LQB demonstrates significant anticonvulsant potential by modulating neurotransmitter pathways, decreasing neuronal excitability, and providing neuroprotection, making it a promising candidate for epilepsy management. Its ability to interact with sodium channels suggests a possible mechanism similar to established anticonvulsant drugs, which could enhance therapeutic options. However, despite promising preclinical findings, the absence of clinical trials limits its immediate applicability. These studies are required to assess its pharmacokinetics, bioavailability, and metabolism in humans, along with long‐term safety evaluations. Additional research is essential to refine dose optimization, assess potential drug interactions, and evaluate efficacy across various seizure types, ensuring its clinical relevance.

Overall findings suggest that LQB showed a moderate BA (–5.4 kcal/mol) to VGSCs in molecular docking studies compared to CBZ (–8.9 kcal/mol); In Vivo results demonstrated LQB‐10 exerts significant (*p* < 0.05) anticonvulsant effects. This disparity suggests that LQB‐10 may exert its effects through non‐competitive or complementary mechanisms, such as indirect VGSC modulation, allosteric interactions, or synergism with CBZ. Our findings highlight that LQB‐10 significantly delays seizure onset, reduces convulsion frequency, and shortens seizure duration in PTZ‐induced models. Notably, the CBZ‐80+LQB‐10 combination exhibited the strongest anticonvulsant activity, indicating a synergistic effect that enhances therapeutic outcomes. These results underscore the importance of integrating In Vivo and In Silico approaches to better understand the pharmacological profile of candidate compounds and support further investigation, including structural optimization and molecular dynamics simulations.

Although LQB exhibited promising anticonvulsant effects, possibly via VGSC modulation, caution must be taken as VGSCs are ubiquitously expressed in both the central and peripheral nervous systems (de Lera Ruiz and Kraus 2015). In particular, their role in regulating peristaltic movement suggests that systemic administration of VGSC inhibitors may cause gastrointestinal disturbances (Coates et al. 2019). This aligns with earlier findings that non‐selective sodium channel blockers can impair smooth muscle function. Thus, despite its efficacy, LQB's therapeutic use as an antiepileptic agent may be limited without further modification or brain‐targeted delivery strategies. In addition, despite its promising results, the study's limitations include a small sample size, a lack of long‐term evaluation, and the need for validation across diverse seizure models, requiring further research for clinical translation.

## 5 Conclusion

In conclusion, LQB exhibited anticonvulsant effects in PTZ‐induced convulsions in chicks, likely through its modulation of VGSCs. The results indicate that LQB at 10 mg/kg significantly (*p* < 0.05) increased seizure latency (62.20 ± 4.31 sec), reduced seizure frequency (15.20 ± 1.43), and decreased seizure duration (127.20 ± 3.52 sec) compared to the control group. Moreover, In Silico studies revealed that LQB moderately binds to VGSCs with a binding energy of −5.4 kcal/mol, forming multiple HBs that support its role in seizure modulation. Together, these findings suggest that LQB may exert its anticonvulsant effects through interaction with VGSC. Further research is needed to confirm LQB's mechanism of action, optimize its structure, and validate its therapeutic potential through molecular dynamics simulations, in vitro, and In Vivo studies supporting information.

## Author Contributions

**Asifa Asrafi**: conceptualization, writing ‐ original draft, writing ‐ review and editing. **Mohammad Aslam**: conceptualization, writing ‐ original draft, writing ‐ review and editing, formal analysis, project administration. **Ali G. Alkhathami**: conceptualization, writing ‐ original draft, writing ‐ review and editing, formal analysis, project administration, resources, supervision. **Md. Sakib Hossain**: writing ‐ original draft, writing ‐ review and editing. **Imam Hossen Rakib**: investigation, methodology, formal analysis, resources, software. **Md. Sakib Al Hasan**: conceptualization, writing ‐ original draft, writing ‐ review and editing, formal analysis, supervision. **Feroz Khan Nun**: investigation, methodology, writing ‐ original draft, software. **Md. Faisal Amin**: software, resources, visualization. **Muhammad Torequl Islam**: conceptualization, investigation, writing ‐ original draft, writing ‐ review and editing, visualization, validation, methodology, software, formal analysis, project administration, resources, supervision, data curation.

## Ethics Statement

This study was approved by the Animal Ethics Committee of Khulna University (KUAEC‐2024‐03‐01).

## Conflicts of Interest

The authors declare no conflicts of interest.

## Peer Review

The peer review history for this article is available at https://publons.com/publon/10.1002/brb3.70675

## Supporting information




**Supporting material**: brb370675‐sup‐0001‐LQB‐convulsion‐data.xlsx

## Data Availability

Data will be made available on request.
